# Novel intravascular tantalum oxide-based contrast agent achieves improved vascular contrast enhancement and conspicuity compared to Iopamidol in an animal multiphase CT protocol

**DOI:** 10.1186/s41747-024-00509-2

**Published:** 2024-10-04

**Authors:** Maurice M. Heimer, Yuxin Sun, Sergio Grosu, Clemens C. Cyran, Peter J. Bonitatibus, Nikki Okwelogu, Brian C. Bales, Dan E. Meyer, Benjamin M. Yeh

**Affiliations:** 1 Department of Radiology and Biomedical Imaging, University of California, San Francisco, CA USA; 2 Department of Radiology, University Hospital, LMU Munich, Munich, Germany; 3 Rensselaer Polytechnic Institute, Saratoga Springs Troy, Troy, NY USA; 4 GE HealthCare Technology & Innovation Center, Niskayuna, NY USA

**Keywords:** Computed tomography angiography, Contrast media, Models (animal), Nanoparticles, Tantalum oxide

## Abstract

**Background:**

To assess thoracic vascular computed tomography (CT) contrast enhancement of a novel intravenous tantalum oxide nanoparticle contrast agent (carboxybetaine zwitterionic tantalum oxide, TaCZ) compared to a conventional iodinated contrast agent (Iopamidol) in a rabbit multiphase protocol.

**Methods:**

Five rabbits were scanned inside a human-torso-sized encasement on a clinical CT system at various scan delays after intravenous injection of 540 mg element (Ta or I) per kg of bodyweight of TaCZ or Iopamidol. Net contrast enhancement of various arteries and veins, as well as image noise, were measured. Randomized scan series were reviewed by three independent readers on a clinical workstation and assessed for vascular conspicuity and image artifacts on 5-point Likert scales.

**Results:**

Overall, net vascular contrast enhancement achieved with TaCZ was superior to Iopamidol (*p* ≤ 0.036 with the exception of the inferior vena cava at 6 s (*p* = 0.131). Vascular contrast enhancement achieved with TaCZ at delays of 6 s, 40 s, and 75 s was superior to optimum achieved Iopamidol contrast enhancement at 6 s (*p* ≤ 0.036. Vascular conspicuity was higher for TaCZ in 269 of 300 (89.7%) arterial and 269 of 300 (89.7%) venous vessel assessments, respectively (*p* ≤ 0.005), with substantial inter-reader reliability (κ = 0.61; *p* < 0.001) and strong positive monotonic correlation between conspicuity scores and contrast enhancement measurements (*ρ* = 0.828; *p* < 0.001).

**Conclusion:**

TaCZ provides absolute and relative contrast advantages compared to Iopamidol for improved visualization of thoracic arteries and veins in a multiphase CT protocol.

**Relevance statement:**

The tantalum-oxide nanoparticle is an experimental intravenous CT contrast agent with superior cardiovascular and venous contrast capacity per injected elemental mass in an animal model, providing improved maximum contrast enhancement and prolonged contrast conspicuity. Further translational research on promising high-*Z* and nanoparticle contrast agents is warranted.

**Key Points:**

- There have been no major advancements in intravenous CT contrast agents over decades.
- Iodinated CT contrast agents require optimal timing for angiography and phlebography.
- Tantalum-oxide demonstrated increased CT attenuation per elemental mass compared to Iopamidol.
- Nanoparticle contrast agent design facilitates prolonged vascular conspicuity.

**Graphical Abstract:**

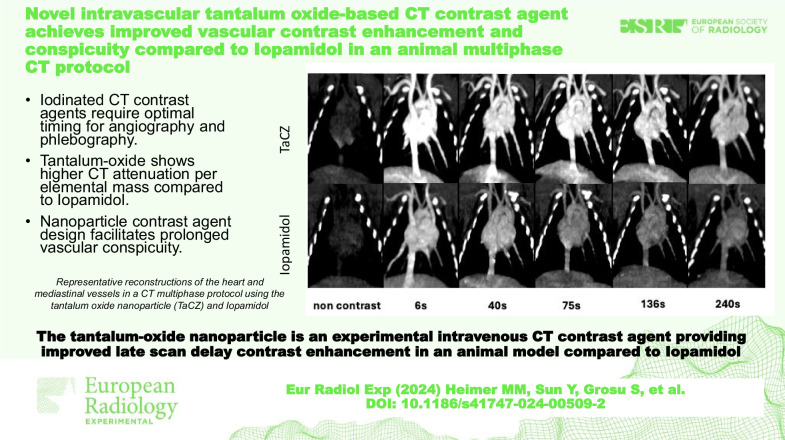

## Background

In recent decades, major advancements in computed tomography (CT) technology, software, and image reconstruction algorithms have helped increase temporal and spatial resolution and have ultimately improved diagnostic quality in non-invasive imaging of vasculature in dedicated CT angiographies and regular body scans [1–4]. At the same time, except for the optimization of contrast delivery protocols, developments in alternative non-iodine CT contrast materials have been lacking over the past several decades [5, 6]. Currently, iodinated contrast agents remain the only Food and Drug Administration-approved and available intravenous contrast materials for CT with a reported hypersensitivity reaction prevalence of 0.73% [7].

Previous preclinical studies of contrast materials incorporating high-*Z* elements and those acting as blood pool agents have been shown to improve vascular CT imaging [8–10]. Recently, a novel carboxybetaine zwitterionic tantalum oxide (TaCZ) nanoparticle (*Z* = 73, k-edge = 67.4 keV) was described as a promising intravenous contrast agent with potentially clinically feasible physicochemical, biological, and CT imaging properties [5, 11, 12]. A prior porcine preclinical study revealed superior large blood vessel and hepatic contrast enhancement of TaCZ in an abdominal CT protocol compared to an iodinated contrast agent, which to date has not been validated in a small-size-vessel multiphase CT protocol [12].

The objective of our study was to assess TaCZ *versus* a conventional clinical iodinated contrast agent for vascular contrast enhancement of thoracic arteries and veins in a non-gated rabbit multiphase CT study.

## Methods

### Animals

Animal experiments were approved by the Veterans Health Administration (VA) San Francisco Institutional Animal Care and Use Committee and conducted in accordance with National Institutes of Health and US Department of Agriculture (USDA) guidelines. Five female New Zealand white rabbits (Western Oregon Rabbit Company, Philomath, OR, USA) with a weight of 4.1 ± 0.3 kg (mean ± standard deviation), ranging from 3.6 kg to 4.4 kg, underwent two consecutive scans with different contrast agents as follows.

Prior to imaging, anesthesia was induced and maintained to effect with isoflurane at 1–3% vol in 2 L/min oxygen flow. A 24-gauge catheter was placed in the marginal ear vein for intravenous contrast material administration. TaCZ (240 mg of tantalum/mL) formulated as previously described [11], or Iopamidol (Isovue-300 mg, 300 mg of iodine/mL, Bracco, Milan, Italy) were manually injected at dose concentrations of 540 mg active element per kg animal weight over 8 s, followed by 3 mL of saline flush. Imaging with the alternative contrast agent was repeated during the first injection. Completeness of washout was confirmed on non-contrast imaging prior to initiating the second contrast exam. The order of contrast agent injection was randomized, such that half the rabbits received each agent first. Animals were allowed to recover from anesthesia between the two exams.

### Encasements

**Fig. 1:**
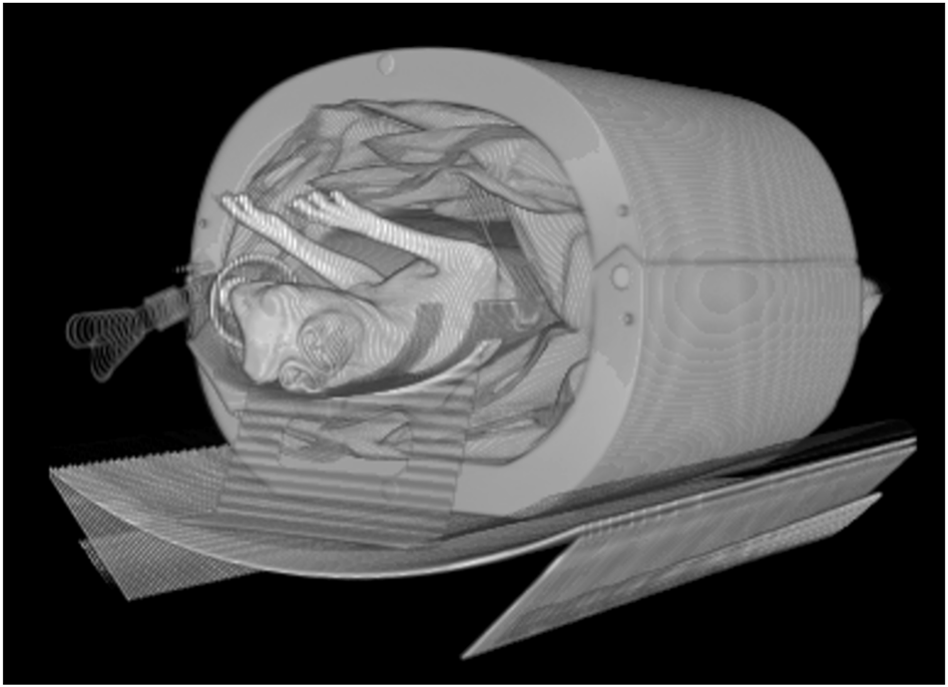
CT volume-rendered reconstruction showcasing the experimental setup. Rabbits were placed supine on a customized carbon sled and centered in a custom-made adipose-equivalent plastic encasement to simulate human size and attenuation. Polyethylene bags containing canola oil were used to fill in the air gaps

A custom-made adipose-equivalent plastic encasement (Computerized Imaging Reference Systems, Norfolk, VA, USA) of 102 cm circumference and 30 cm length was used to simulate the mass and attenuation of a human torso. Animals were placed on a customized carbon sled (Fibreworks Composites, Mooresville, NC, USA) and centered within the encasement in the supine position. Polyethylene bags containing canola oil were placed around the encasement to fill in the air gaps with fat-equivalent material (Fig. 1).

### Imaging

Animals were scanned on a clinical CT scanner (IQon, Philipps, Best, the Netherlands) using a standard oncology body protocol (helical mode with 1.2 pitch, 40-mm collimation, 0.4-s gantry rotation time, 120 kVp, 200 mA, CT dose index volume 18.1) to resemble clinical CT image quality acquired in human studies. The torso of the animals was scanned in a single 30-cm scan to allow imaging from the neck to the proximal hind limbs (*z*-axis length of the encasements). For each contrast injection, precontrast scans were acquired before contrast administration, followed by scans at delays of 6 s, 40 s, 75 s, 136 s, and 240 s after completion of the injection. Images were reconstructed with a standard 41-cm field of view and 1.5-mm slice thickness to facilitate improved vessel delineation using iterative and a standard soft kernel reconstruction algorithm.

### Objective image quality assessment

One reader with 7 years of experience in animal imaging and 4 years in clinical radiology (M.M.H.) reviewed all acquired image series and placed standardized regions of interest (ROIs) in a total of four arterial (ascending aorta, aortic arch, descending aorta, pulmonary trunk) and four venous thoracic vessels (superior and inferior vena cava, subclavian vein, and internal jugular vein)—where appropriate veins were assessed contralateral to the injection site) on a clinical Picture Archiving and Communication System workstation (Visage Imaging, version 7.1.17, San Diego, CA, USA). The maximum axial diameter of the assessed vessels was measured on the aforementioned ROIs at 40 s. For objective assessment, only vessels > 3 mm in diameter were included to ensure reliable measurements. Three ROIs were placed in consecutive image slices per vessel and contrast phase. Net vascular contrast enhancement was assessed by subtraction of corresponding non-contrast CT image ROI values. Image noise was recorded as the standard deviation in ROIs placed in the canola oil bags. Absolute and relative contrast enhancement values were calculated for individual studies; relative values were obtained after normalization to the peak contrast enhancement in the corresponding vessel by either TaCZ or Iopamidol:

$$	{{{{\rm{relative}}}}\; {{{\rm{contrast}}}}\; {{{\rm{enhancement}}}}}_{{{{\rm{agent}}}}} = \\ 	 = ({{{{\rm{measured}}}}\; {{{\rm{attenuation}}}}}_{{{{\rm{agent}}}}} \, {{{\rm{minus}}}}\; {{{\rm{noncontrast}}}}\; {{{\rm{attenuation}}}})/ \\ 	 \quad \, {{{\rm{peak}}}}\; {{{\rm{attenuation}}}}_{{{{\rm{agent}}}}}.$$

### Subjective image quality assessment

**Fig. 2:**
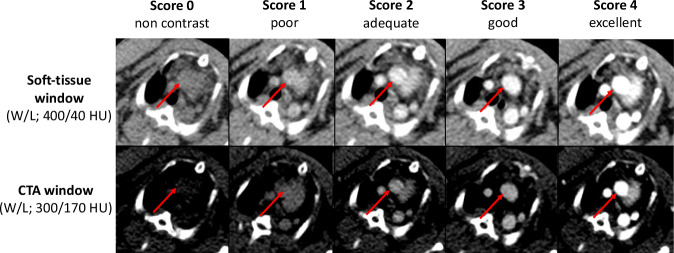
Representative vascular conspicuity scores demonstrated at the upper mediastinal level. Vascular conspicuity scores were demonstrated by representative axial images at the upper mediastinal level. Vascular conspicuity is subjectively graded as non-contrast, poor, adequate, good, and excellent (from left to right); corresponding images are demonstrated at soft tissue (top row; width/level (W/L) = 400/40 HU) and CTA window levels (bottom row; W/L; 300/170 HU). The ascending aorta is indicated with a red arrow. CTA, Computed tomography angiography; L, Level; W, Width

Three radiologists with different levels of experience in clinical radiology (M.M.H., 4 years; S.G., 7 years; and C.C.C., 17 years) reviewed all individually randomized contrast-enhanced image series and were free to adjust CT window levels to preference. Readers graded the overall vascular conspicuity of four arterials (ascending aorta, carotid arteries, axillary artery, pulmonary trunk) and four thoracic venous vessels (superior vena cava, axillary vein, subclavian and internal jugular vein) at each time point representing a variety of different caliber vessels of 2–7 mm on a 5-point Likert scale (0 = non-enhanced, not diagnostic; 1 = poor, confidence reduced; 2 = adequate to exclude major pathology; 3 = good, with high confidence; 4 = excellent). Representative images are shown in Fig. 2. In contrast to the objective assessment, smaller vessels were included that were too small for robust ROI measurements. Additionally, raters scored contrast- and motion-associated artifacts subjectively on a 5-point Likert scale (0 = none; 1 = slight, no relevance for diagnosis; 2 = definite, sufficient for diagnosis; 3 = marked, diagnostic quality reduced; 4 = severe, non-diagnostic).

### Statistical analyses

SPSS (IBM SPSS, version 29.0, Armonk, NY, USA) was used for statistical testing. Descriptive data were expressed as mean ± standard deviation or median where appropriate. For statistical testing, the Wilcoxon Signed Rank Test was used for dependent samples, and the Mann–Whitney *U*-test for independent samples. Statistical significance was defined as *p* < 0.050 in two-tailed hypothesis tests. The Fleiss κ was used for inter-reader reliability. Spearman rank correlation test was used to assess the relationship between paired objective and subjective findings.

## Results

### Objective image quality assessment

**Fig. 3:**
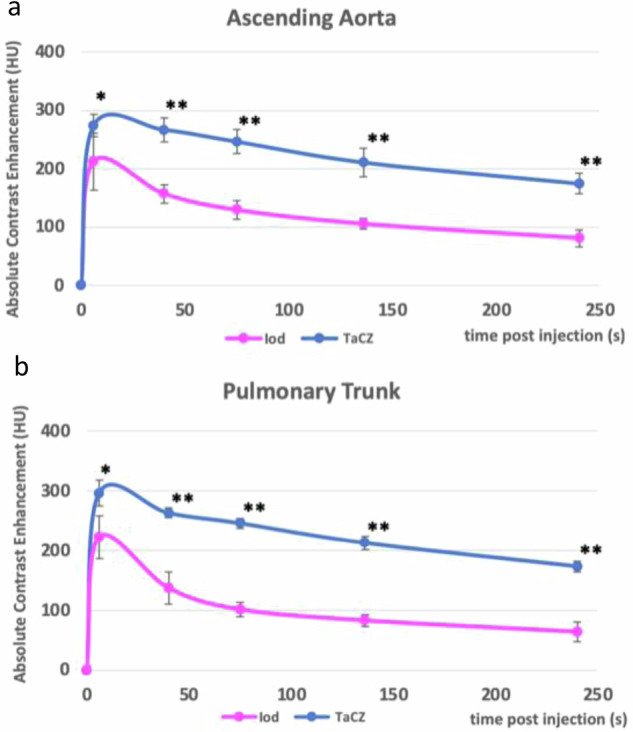
Contrast enhancement time curves for Iopamidol and TaCZ. Measured vascular contrast enhancement achieved in a multiphase CT study. Two representative vessels are displayed: the ascending aorta (**a**) and the pulmonary trunk (**b**). TaCZ (blue) provides higher vascular contrast enhancement compared to Iopamidol (magenta) at all time points (*p < 0.01; **p < 0.001). TaCZ provides superior absolute vascular contrast enhancement in all shown vessels at 75 s compared to Iopamidol at 6 s. Iod, Lopamidol; TaCZ, Carboxybetaine zwitterionic tantalum oxide

Overall, TaCZ displayed increased net vascular attenuation compared to Iopamidol at all assessed time points (Tables 1 and 2). A nonsignificant difference was observed in the inferior vena cava at 6 s (*p* = 0.131). The peak vascular contrast enhancement was obtained at 6 s after contrast administration of both TaCZ and Iopamidol in all assessed vessels. Representative net contrast attenuation curves for the ascending aorta and the pulmonary trunk are displayed in Fig. 3a, b).

**Table 1:** Net contrast enhancement in arterial vasculature for Iopamidol and TaCZ

	Ascending aorta			Aortic arch			Descending aorta			Pulmonary trunk		
Contrast delay, (s)	Iopamidol, (HU)	Tantalum oxide, (HU)	Increase for tantalum oxide compared with Iopamidol, (%)	Iopamidol, (HU)	Tantalum oxide, (HU)	Increase for tantalum oxide compared with Iopamidol, (%)	Iopamdiol, (HU)	Tantalum oxide, (HU)	Increase for tantalum oxide compared with Iopamidol, (%)	Iopamidol, (HU)	Tantalum oxide, (HU)	Increase for tantalum oxide compared with Iopamidol, (%)
6	213 ± 49	274 ± 19	29*	217 ± 66	263 ± 19	21*	207 ± 56	264 ± 21	28*	223 ± 36	296 ± 21	33*
40	158 ± 16	322 ± 21	104**	145 ± 15	265 ± 9	83**	144 ± 23	244 ± 19	69**	138 ± 27	263 ± 8	91**
75	130 ± 16	302 ± 21	132**	119 ± 14	240 ± 7	102**	105 ± 9	229 ± 12	118**	102 ± 13	246 ± 8	141**
136	106 ± 10	266 ± 24	151**	93 ± 7	217 ± 14	133**	90 ± 9	198 ± 13	120**	83 ± 10	213 ± 10	157**
240	82 ± 15	229 ± 17	179**	73 ± 11	236 ± 14	223**	64 ± 8	164 ± 21	156**	64 ± 17	174 ± 11	172**

Unless otherwise indicated, data are mean ± standard deviation obtained in various contrast delays in the ascending aorta, the aortic arch, the descending aorta, and the pulmonary trunk. The asterisk (*) denotes a significant difference of *p* < 0.05 or (**) *p* < 0.001 between the two contrast agents

*HU* Hounsfield units, *TaCZ* Carboxybetaine zwitterionic tantalum oxide nanoparticle

**Table 2:** Net contrast enhancement in venous vasculature for Iopamidol and TaCZ

	Superior vena cava			Inferior vena cava			Subclavian vein			Internal jugular vein		
Contrast delay, (s)	Iopamidol, (HU)	Tantalum oxide, (HU)	Increase for tantalum oxide compared with Iopamidol, (%)	Iopamidol, (HU)	Tantalum oxide, (HU)	Increase for tantalum oxide compared with Iopamidol, (%)	Iopamidol, (HU)	Tantalum oxide, (HU)	Increase for tantalum oxide compared with Iopamidol, (%)	Iopamidol, (HU)	Tantalum oxide, (HU)	Increase for tantalum oxide compared with Iopamidol, (%)
6	211 ± 51	307 ± 31	45*	233 ± 54	237 ± 36	2	183 ± 28	280 ± 36	53**	233 ± 65	313 ± 42	34*
40	127 ± 20	255 ± 14	101**	178 ± 19	240 ± 12	35**	111 ± 14	254 ± 10	129**	125 ± 29	258 ± 13	106**
75	96 ± 21	239 ± 16	149**	109 ± 16	248 ± 40	128**	86 ± 11	236 ± 9	174**	100 ± 27	242 ± 11	142**
136	79 ± 14	196 ± 11	148**	91 ± 19	201 ± 13	121**	67 ± 14	205 ± 14	206**	76 ± 21	210 ± 12	176**
240	49 ± 11	169 ± 13	245**	66 ± 13	144 ± 13	118**	54 ± 11	170 ± 14	215**	58 ± 19	182 ± 10	214**

Unless otherwise indicated, data are mean ± standard deviation obtained in various contrast delays in the superior vena cava, the inferior vena cava, the subclavian vein, and the internal jugular vein. The asterisk (*) denotes a significant difference of *p* < 0.05 or (**) for *p* < 0.001 between the two contrast agents

*HU* Hounsfield Units, *TaCZ* Carboxybetaine zwitterionic tantalum oxide nanoparticle

In the TaCZ cohort, the highest net vascular contrast enhancement was seen in the superior vena cava with 307 ± 31 HU at 6 s, followed by the pulmonary trunk with 296 ± 21 HU at 6 s, the lowest net contrast enhancement was observed in the inferior vena cava with 144 ± 13 at 240 s. In the Iopamidol cohort, the highest net vascular contrast enhancement was achieved in the jugular vein with 233 ± 65 HU at 6 s, followed by inferior vena cava with 233 ± 54 at 6 s, the lowest net contrast enhancement was observed in the superior vena cava at 49 ± 11 HU at 240 s. Vascular contrast decay between the first and last scan delay was prolonged in the TaCZ cohort compared to Iopamidol in both arterial (-36.3 ± 7.9% *versus* -65.9 ± 8.8%; all *p* < 0.001) and venous vessels (-40.6 ± 8.8% *versus* -73.2 ± 6.5%; all *p* < 0.001). Vessel diameters measured on average: ascending aorta (6 ± 1 mm), aortic arch (5 ± 1 mm), descending aorta (5 ± 1 mm), pulmonary trunk (7 ± 1 mm), superior vena cava (5 ± 1 mm), inferior vena cava (6 ± 2 mm), subclavian vein (7 ± 2 mm), and internal jugular vein (5 ± 1 mm). No significant difference in image noise was observed between contrast agent cohorts (15 ± 1 HU for TaCZ *versus* 14 ± 2 HU for Iopamidol; *p* = 0.101).

### Subjective image quality assessment

**Fig. 4:**
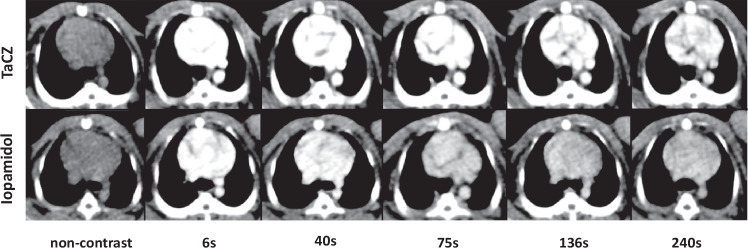
TaCZ improves thoracic vascular contrast with superior conspicuity at soft-tissue window levels. Vascular contrast enhancement at the aortopulmonary level. Representative axial images at 1.5-mm slice thickness before and 6 s, 40 s, 75 s, 136 s, and 240 s after intravenous administration of TaCZ (top row) or Iopamidol (bottom row) at soft-tissue window levels (W/L = 400/40 HU). TaCZ provides superior contrast enhancement at all scan delays with sustained vascular enhancement at 240 s after contrast administration. L, Level; W, Width; TaCZ, Carboxybetaine zwitterionic tantalum oxide

**Fig. 5:**
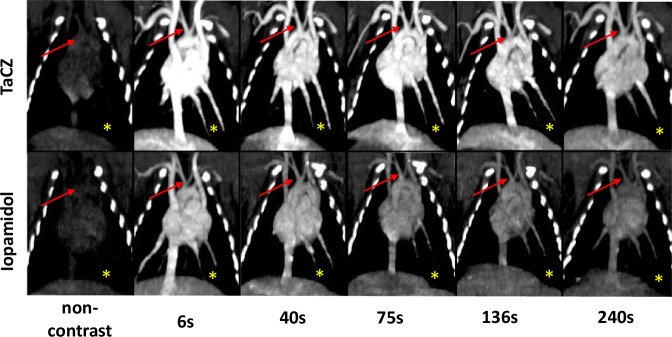
TaCZ improves thoracic vascular contrast with superior conspicuity at CTA window levels. Vascular contrast enhancement of the heart and large thoracic vasculature. Representative coronal maximum intensity projection at 10 mm before and 6 s, 40 s, 75 s, 136 s, and 240 s after intravenous administration of TaCZ (top row) or Iopamidol (bottom row) at CTA window levels (W/L = 300/170 HU). The heart and larger thoracic vessels are displayed; the brachiocephalic trunk (red arrow) and left inferior pulmonary artery and vein (yellow asterisk) are highlighted. Overall, TaCZ provides superior contrast enhancement at all scan delays with sustained vascular enhancement at 240 s after contrast administration. Parenchymal liver enhancement is also increased in the TaCZ cohort. CTA, Computed tomography angiography; L, Level; TaCZ, Carboxybetaine zwitterionic tantalum oxide; W, Width

**Fig. 6:**
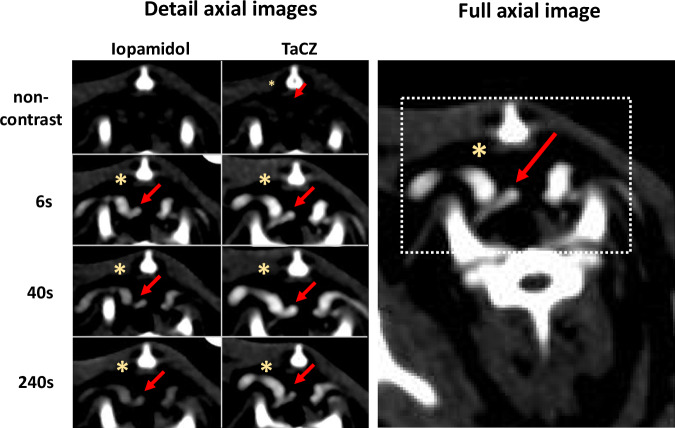
TaCZ improves the conspicuity of small arteries and veins at late contrast delays. Vascular contrast enhancement at the level of the thoracic aperture. The vascular ROI is enclosed by a white dotted rectangle (right image). Representative detail axial images (left) of 1.5 mm slice thickness display vascular contrast enhancement of the bilateral subclavian veins (yellow asterisk—right subclavian vein) at their confluence with the internal jugular veins. The brachiocephalic artery is highlighted by a red arrow, the left carotid artery is obscured by the vein. Images are displayed at CTA window levels (W/L = 300/170 HU) before (non-contrast), 6 s, 40 s, and 240 s after contrast administration (Iopamidol, left column; TaCZ, right column). TaCZ provides higher contrast enhancement of the shown vessels at all scan delays with sustained contrast at 240 s. CTA, Computed tomography angiography; L, Level; TaCZ, Carboxybetaine zwitterionic tantalum oxide; W, Width

Overall, interobserver agreement was substantial (κ = 0.61; *p* < 0.001). TaCZ achieved superior conspicuity scores compared to Iopamidol in both arterial and venous vasculature as displayed in Figs. 4–6 providing soft-tissue and CT angiography (CTA) window levels. At 6 s, vascular conspicuity of all vessels was rated higher for the TaCZ cohort compared to Iopamidol (on median excellent *versus* good; *p* ≤ 0.005). At 240 s, the difference was accentuated in favor of TaCZ (on median good *versus* poor; all *p* < 0.001). Overall, the conspicuity score achieved with TaCZ was graded higher than Iopamidol in 269 of 300 (89.7%) arterial and 269 of 300 (89.7%) venous vessel assessments, respectively (*p* ≤ 0.005).

When grouping data by contrast delay, TaCZ displayed superior vascular conspicuity in 60 of 100 (60.0%) of assessed vessels at 6 s, 87 of 100 (87.0%) at 40 s, 99 of 100 (99.0%) at 75 s, and 200 of 200 (100%) at 136 to 240 s. Conversely, Iopamidol was not rated superior in any of the assessed studies. Significant differences in vascular conspicuity between differently sized vessels were observed in the Iopamidol group at late contrast enhancement; the perceived vascular conspicuity of the axillary artery (~1–2 mm) was significantly lower as all of the other arterial and venous vessels at 136 s (*p* ≤ 0.001). and at 240 s (all; *p* < 0.001). No conspicuity difference between differently sized vessels was observed in the TaCZ cohort. No contrast- or motion-related artifacts were observed between the TaCZ and Iopamidol cohort (0 *versus* 0, *p* = 0.772; and 0 *versus* 0, *p* = 0.363, respectively). Overall, there was a strong positive monotonic correlation between matched vascular contrast enhancement and perceived vascular conspicuity scores (*ρ* = 0.828; *p* < 0.001).

## Discussion

Our study aimed to assess the enhancement obtained with TaCZ compared to that allowed by a clinical iodinated contrast agent in a medium-sized animal imaged in a human torso-sized adipose equivalent encasement using a clinical scanner. The assessed vessel calibers ranged from 2 mm to 8 mm, which are similar to the size of human coronary arteries and small veins. Our findings showed that TaCZ provides increased and prolonged vascular contrast enhancement compared to Iopamidol, with a strong positive correlation between net contrast enhancement and conspicuity scores. Our results are in line with a previous study that showed 46% higher CT image contrast per elemental contrast mass of tantalum compared to Iopromide and improved abdominal vascular contrast enhancement and conspicuity in a porcine multiphase CT protocol at different girths [8, 12]. While at early scan delays Iopamidol also achieved good or excellent contrast enhancement, Iopamidol vascular conspicuity was rated as poor overall at late contrast delays compared with TaCZ (*p* < 0.001). Our study builds on the results of this prior publication by studying a different animal species, smaller vessels, and a different iodine contrast agent comparator, and focusing on the cardiopulmonary rather than abdominal vessels.

A principal limitation of iodinated contrast media is the comparatively low absorption coefficient for higher energy x-rays in the CT imaging spectrum, which results in a reduced attenuation per molar mass and per gram, particularly in the case of obese body habitus where beam hardening is prominent [10, 12, 13]. Also, due to some similarity in spectral attenuation characteristics between iodine and calcium, the material differentiation, evaluation, and quantification of calcified tissues, such as atherosclerotic plaques or valves, remains inaccurate even with optimized algorithms used with dual-energy CT equipment [10, 14, 15]. Sartoretti et al [9] showed that elements with higher atomic numbers allow for better discrimination between contrast-filled vessel lumens and calcified plaques when compared to Iopamidol at multi-energy CT. This property of high-*Z* contrast agents resulted in superior diagnostic quality for varying degrees of stenosis and lower image noise in virtual non-contrast images, promising favorable applications in spectral imaging [9, 16].

Another major limitation of available clinical iodinated contrast agents is their rapid, diffusion-driven wash-in and wash-out between vascular and interstitial compartments (*i.e*., pharmacokinetic distribution) due to their small molecular size [10, 13]. In consequence, diagnostic scan delay windows are brief and may require test-bolus or bolus-tracking to achieve acceptable image quality in a variety of cardiovascular protocols [13]. Despite these efforts, up to 4–27% of vascular studies show an unsatisfactory or even insufficient vascular contrast enhancement for key vascular structures, particularly in arteries with slow flow or in veins, resulting in a reduced diagnostic certainty or even requiring repeat imaging [17, 18]. Unlike current clinical agents (molecular size ~1–2 nm), the somewhat larger particle size (~3–6 nm) of nanoparticle-based CT contrast agents may provide longer blood pool dwell times after intravenous injection [13]. The longer vascular enhancement of TaCZ may allow for more relaxed timing of CT scanning after injection, slower and thus presumably safer contrast injection rates, and more reliable complete filling of the entire vasculature, including branches with slower flow and veins, compared to the current iodinated contrast agents.

Beyond cardiovascular imaging, the majority of contrast-enhanced CT studies are performed with venous phase scan delays. While differentiation of soft tissues and lesion detection is the priority, vascular disease delineation remains important and, if discovered, may require immediate treatment. Several studies have reported on the prevalence of incidental venous thromboembolism occurring in 1.1–7.3% of oncology patients [19–23], and up to 31.9% in high-risk intensive care patients [24]. A relevant proportion of venous thromboembolism is missed on venous contrast CT as compared to dedicated vascular protocols [25]. Our findings suggest that TaCZ could provide both improved arterial and venous vascular contrast enhancement in a single venous phase CT scan phase due to prolonged vascular enhancement [12]. A small series of preclinical studies have shown that experimental long-circulating CT contrast agents facilitate improved detection of venous thromboembolism up to 24 h after contrast injection [13, 26, 27]. However, further investigation is needed to assess the role and value of experimental contrast agents beyond cardiovascular imaging with correlation to pathology.

Several limitations of our study require consideration. Firstly, only vascular contrast enhancement and conspicuity were assessed in a variety of arterial and venous vessels in a medium-sized animal model multiphase CT protocol; the detection and accuracy of diagnosing vascular pathology were not evaluated. Secondly, our imaging studies were non-gated to the cardiac cycle, which is a prerequisite for standard cardiac and aortic CT protocols. Nonetheless, our results show that TaCZ provides improved and sustained vascular contrast enhancement to provide CTA equivalent vascular contrast in early and late venous contrast delays. Thirdly, a standard concentration of 240 mg active element per mL was used in this experiment, which lies at the lower range of iodinated contrast agents used in clinical practice [5, 11, 13]. Moreover, images were acquired using a standard thoracic CT protocol at 120 kVp. Dedicated cardiovascular protocols are routinely acquired at lower kVp settings, which improve the performance of Iopamidol [28, 29]. However, our results remain relevant to the vast majority of CT exams at 120 kVp and require further assessment of both contrast agents at different tube settings. Additionally, while the sample size was limited to five rabbits, the observed differences in contrast enhancement between the contrast materials were substantial enough to demonstrate statistical significance. Also, rabbit anatomy and cardiovascular circulation are different from human circulation with significantly smaller vessel size and faster circulation time in rabbits, which accelerates contrast pharmacodynamics and vascular dilution rate compared to humans. Accelerated circulation time and the contrast material delivery protocol may explain the early vascular contrast equilibrium seen between arteries and veins. Nevertheless, multiple scan delays were studied including late arterial, venous, and late venous contrast delays. Lastly, the safety profile of novel contrast materials is unknown and requires further investigation in human studies.

In conclusion, TaCZ provides superior peak and prolonged arterial and venous contrast enhancement compared to Iopamidol in a rabbit multiphase CT protocol. The magnitude of relative contrast material advantage increased over time and was largest at 240 s, the latest time point studied. Our findings suggest that TaCZ may especially improve vascular assessment at venous scan delays. Further translational research for the clinical potential of TaCZ is warranted.

## Data Availability

The datasets used and/or analyzed during the current study are available from the corresponding author upon reasonable request.

## Abbreviations

CT: Computed tomography
CTA: CT angiography
ROI: Region of interest
TaCZ: Carboxybetaine zwitterionic tantalum oxide
W/L: Width/level
